# Neurological research and practice: the first year

**DOI:** 10.1186/s42466-020-0054-9

**Published:** 2020-02-28

**Authors:** Werner Hacke

**Affiliations:** University of Heidelberg, Department of Neurology, Im Neuenheimer Feld 400, 69120 Heidelberg, Germany

One year ago, the first articles in *Neurological Research and Practice* (NRP), a new peer-reviewed open access online-only scientific Journal were published. *Neurological Research and Practice* is the official Journal of the German Neurological Society (Deutsche Gesellschaft für Neurologie (DGN)), one of the largest national neurological societies worldwide with more than 10,000 members. NRP was created by the leadership of the DGN as an international, English language publication with a broad thematic scope reflecting all clinical, translational and basic research aspects of neurology and neuroscience. We strive to provide a forum for clinicians and scientists working in all areas of neurology including, but not limited to, genetics, vascular diseases, critical care neurology, disorders of the spine, movement disorders, neuroimmunology, infections, neurooncology, epilepsy, neuroimaging and neuroradiology, and degenerative disorders. The Journal publishes Research Articles, Reviews, Clinical Trial Protocols, Guidelines, Standard Operating Procedures, Historical Articles and Reviews and Letters to the Editor.

The announcement of the new Journal came at a time of great competition between neurology journals [1]. With the increasing numbers of journals offering different publishing options for authors, some of which are of quality and some so-called predator journals, it can be difficult to establish a serious, quality- oriented open access journal in this environment. One of the characteristics that distinguishes NRP from many other new journals is that we believe in a strict peer-review process and unlike other open access options we currently do not charge any publication fees, this cost is covered by the DGN.

After 18 months of preparatory work, the first set of seven articles were published by the end of February 2019. Now, one year later, it seems to be a good time to review and reflect on the achievements of the first year of NRP. As the founding Editor-in-Chief of NRP, I am currently assisted by a most prominent group of almost 100 international Editorial Board Members, among them more than 30% are women. Not surprisingly about 50% of them represent the German Neurological Society, but the remaining experts are geographically diverse and as Editor in Chief I strive to grow the Journal network internationally. We have appointed Section Editors for the topics of History of Neurology, Standard Operating Procedures, and Guidelines. A board of more than 100 reviewers with experience in all areas of neurology and its sister disciplines has been assembled. With such a strong network of neurologists based in Germany it is no surprise that most of our content currently originates from all parts of Germany, from universities and large teaching hospitals. We are pleased to see submissions from elsewhere in Europe but are aiming to grow submissions from all areas, worldwide.

In the first 12 months since launch, more than 40 articles have been published. Most of the Reviews were by invitation, but not all those submitted were accepted. There is a stringent and detailed peer-review process for most articles published in the Journal. Clinical Trial Protocols of trials that are publicly funded, and have already undergone an exhaustive review process, and translations of specific guidelines that are approved by one or several scientific societies and already published in an extensive German version are accepted without additional peer review, with assessment by the Editor in Chief. Unofficial recommendations and Trial Protocols that are not publicly funded undergo a regular peer review process, just like research papers and other articles. Concerning official guidelines, we follow a policy of only publishing abridged versions of already published German guidelines in areas that are not yet covered by other recent international guidelines or that cover an area specific to German neurology.

Some of the manuscripts published have already been cited and several of the papers have already been downloaded more than 1000 times, which is an achievement for a new journal. At the time of writing this, among the 10 most accessed articles were 5 Reviews, 3 Research Articles, 1 Guideline and 1 Standard Operating Procedure [2–11]. Most of them have been accessed more than 1000 times (Table 1). So, this is what has been achieved in the first 12 months; what are our plans for the next year?

**Table 1:** Articles with highest access rates and citations

Article (Author, title)	Reference	Type	Article accesses^a^	Citations/Altimetric Score^b^
**Sembill et al Resumption of oral anticoagulation after spontaneous intracerebral hemorrhage**	Neurol. Res. Pract. 1, 12 (2019) [6] doi:10.1186/s42466-019-0018-0	Review	3133	
**Mokli et al Computer-aided imaging analysis in acute ischemic stroke – background and clinical applications**	Neurol. Res. Pract. 1, 23 (2019) [11] doi:10.1186/s42466-019-0028-y	Review	2661	
**Linker and Chan Navigating choice in multiple sclerosis management**	Neurol. Res. Pract. 1, 5 (2019) [3] doi:10.1186/s42466-019-0005-5	Review	2242	
**Dziewas et al Safety and clinical impact of FEES – results of the FEES-registry**	Neurol. Res. Pract. 1, 16 (2019) [5] doi:10.1186/s42466-019-0021-5	Research Article	2242	5/75
**Göttle et al An unmet clinical need: roads to remyelination in MS**	Neurol. Res. Pract. 1, 21 (2019) doi:10.1186/s42466-019-0026-0	Review	1818	
**Weber et al Distribution and evolution of acute interventional ischemic stroke treatment in Germany from 2010 to 2016**	Neurol. Res. Pract. 1, 4 (2019) [4] doi:10.1186/s42466-019-0010-8	Research Article	1642	5/27
**Ganti et al GCS 15: when mild TBI isn’t so mild**	Neurol. Res. Pract. 1, 6 (2019) [8] doi:10.1186/s42466-018-0001-1	Research Article	1310	
**Bösel First-ever epileptic seizure in adult patients**	Neurol. Res. Pract. 1, 3 (2019) [9] doi:10.1186/s42466-019-0006-4	Standard Operating Procedure	1256	
**Ayzenberg et al General principles and escalation options of immunotherapy in autoantibody-associated disorders of the CNS**	Neurol. Res. Pract. 1, 32 (2019) [10] doi:10.1186/s42466-019-0037-x	Review	1240	
**Diener et al Cryptogenic stroke and patent foramen ovale**	Neurol. Res. Pract. 1, 1 (2019) [2] doi:10.1186/s42466-019-0008-2	Guideline	844	

^a^Article accesses counted as indicated on Neurological Research and Practice on 15 January 2020

^b^Citations and Altmetric Scores as indicated on Neurological Research and Practice on 15 January 2020

Our publication pipeline is small but growing and we continue to invite Reviews on new developments and perspectives, and we welcome submissions of Research Articles. Several Clinical Trial Protocols are already announced, and we have lists of upcoming Standard Operating Procedures and Guidelines that will be published within the next 12 months.

The Editorial Board has been a great resource in assisting with launching and establishing NRP, and we continue to grow the Board. By the end of 2020, we will have the first rotation of our Editorial Board and in 2021, we plan to identify Section Editors for the key areas of neurology such as basic science, neuroimmunology, neurooncology, neurodegeneration and movement disorders, stroke, interventional neurology and critical care, pediatric neurology, cognitive neurology and functional imaging, and neuromuscular disorders.

As the Founding Editor of NRP I am very grateful for the overwhelming support by the leadership of the German Neurological Society represented by our current President Professor Christine Klein, Past President Professor Gereon Fink, Incoming President Christian Gerloff, the Secretary Professor Peter Berlit and the Chief administrative Officer Dr. Thomas Thiekötter, the publisher BMC and all my supporters in the Editorial Board, all the authors for submitting their articles and the group of reviewers for making such an important contribution. It is their work that makes the first year of NRP so successful.

Please continue to support us to make NRP a well-received and influential voice of German neurology publishing content with international relevance.

all the best

Werner Hacke
